# Developing an SNP dataset for efficiently evaluating soybean germplasm resources using the genome sequencing data of 3,661 soybean accessions

**DOI:** 10.1186/s12864-024-10382-3

**Published:** 2024-05-14

**Authors:** Yongchao Niu, Wai-Shing Yung, Ching-Ching Sze, Fuk-Ling Wong, Man-Wah Li, Gyuhwa Chung, Hon-Ming Lam

**Affiliations:** 1 Centre for Soybean Research of the State Key Laboratory of Agrobiotechnology, The Chinese University of Hong Kong, Hong Kong SAR, China; 2 School of Life Sciences, The Chinese University of Hong Kong, Hong Kong SAR, China; 3 Department of Biotechnology, Chonnam National University, Yeosu-Si, Republic of Korea; 4 Shenzhen Research Institute, The Chinese University of Hong Kong, Shenzhen, 518000 China; 5 Institute of Environment, Energy and Sustainability, The Chinese University of Hong Kong, Hong Kong SAR, China

**Keywords:** Soybean, Genome sequencing, Single nucleotide polymorphism, SNP marker, Insertion/deletion, InDel marker, Germplasm evaluation, Large-effect mutation (LEM), Naturally occurring mutant

## Abstract

**Background:**

Single nucleotide polymorphism (SNP) markers play significant roles in accelerating breeding and basic crop research. Several soybean SNP panels have been developed. However, there is still a lack of SNP panels for differentiating between wild and cultivated populations, as well as for detecting polymorphisms within both wild and cultivated populations.

**Results:**

This study utilized publicly available resequencing data from over 3,000 soybean accessions to identify differentiating and highly conserved SNP and insertion/deletion (InDel) markers between wild and cultivated soybean populations. Additionally, a naturally occurring mutant gene library was constructed by analyzing large-effect SNPs and InDels in the population.

**Conclusion:**

The markers obtained in this study are associated with numerous genes governing agronomic traits, thus facilitating the evaluation of soybean germplasms and the efficient differentiation between wild and cultivated soybeans. The natural mutant gene library permits the quick identification of individuals with natural mutations in functional genes, providing convenience for accelerating soybean breeding using reverse genetics.

**Supplementary Information:**

The online version contains supplementary material available at 10.1186/s12864-024-10382-3.

## Background

Single nucleotide polymorphism (SNP) markers are widely applied in crop research and breeding. Among SNP discovery approaches, SNP genotyping arrays are promising tools applied in rice [1], maize [2], and other crops [3–5]. In soybeans, seven moderate- to high-density soybean arrays (8-618 K) have been developed for soybean genome-wide association studies (GWAS), population structure, and domestication studies [6–11]. Although the early soybean SNP arrays had high SNP densities, they were only based on a small number of soybean accessions [7, 8]. Recent soybean SNP arrays have utilized thousands of samples, but the number of wild soybean (*Glycine soja*) germplasms included is still limited [6, 10]. In addition, the high-density SNP array platforms are inconvenient and costly for the preliminary evaluation of soybean germplasm resources. In recent years, with the development of sequencing technologies, studies based on large-scale population resequencing have been published [12–14], making it possible to use soybean genetic resources to screen for highly efficient SNP markers. Cultivated soybeans (*G. max*) are domesticated from wild soybeans (*G. soja*) [15] which provide valuable genetic resources for soybean improvement. Due to the presence of natural and artificial hybridization between wild and/or cultivated soybeans, the identification and utilization of polymorphic markers in wild soybeans, along with markers capable of distinguishing between wild and cultivated soybeans, provide important tools for advancing soybean breeding and cultivation practices. Although some databases provide queries for mutation information, such as soybase.com, soykb.org, and SoyGVD [16], it is still necessary to construct a natural mutant gene library that focuses on large-effect mutations that are validated in multiple populations. The purpose of this study is to use published soybean genomic sequencing data, with a special focus on the selection of wild soybean resources, to obtain highly representative differentiation SNP and insertion/deletion (InDel) markers between wild and cultivated populations for distinguishing wild from cultivated soybeans, evaluating wild or cultivated soybean germplasm resources, and constructing a natural mutation library for soybean breeding or gene function studies. Thus, we selected 763 soybean individuals, including 345 wild and 418 cultivated soybeans published by Kim et al. [13] as the test population, and 2,898 soybean populations (including 103 wild soybeans) published by Liu et al. [12] as the validation population (Table S1). Genomic differentiation regions between wild and cultivated populations were identified for the purpose of screening SNP and InDel markers with good polymorphism between and within the cultivated and wild soybean populations. The SNPs and InDels that occur in the coding sequences are useful for studying the functions of agronomic trait-related genes, and their high degrees of sequence conservation can serve as efficient markers for germplasm resource evaluation. Finally, we researched genes with large-effect mutations (LEM) in the population to construct a natural mutant gene library. This study will provide valuable data to support and facilitate soybean germplasm evaluation, breeding, and reverse genetics research (Fig. S1).

## Results

### Identification of differentiation genomic regions between cultivated and wild soybean populations and SNP screening using the test population

**Fig. 1:**
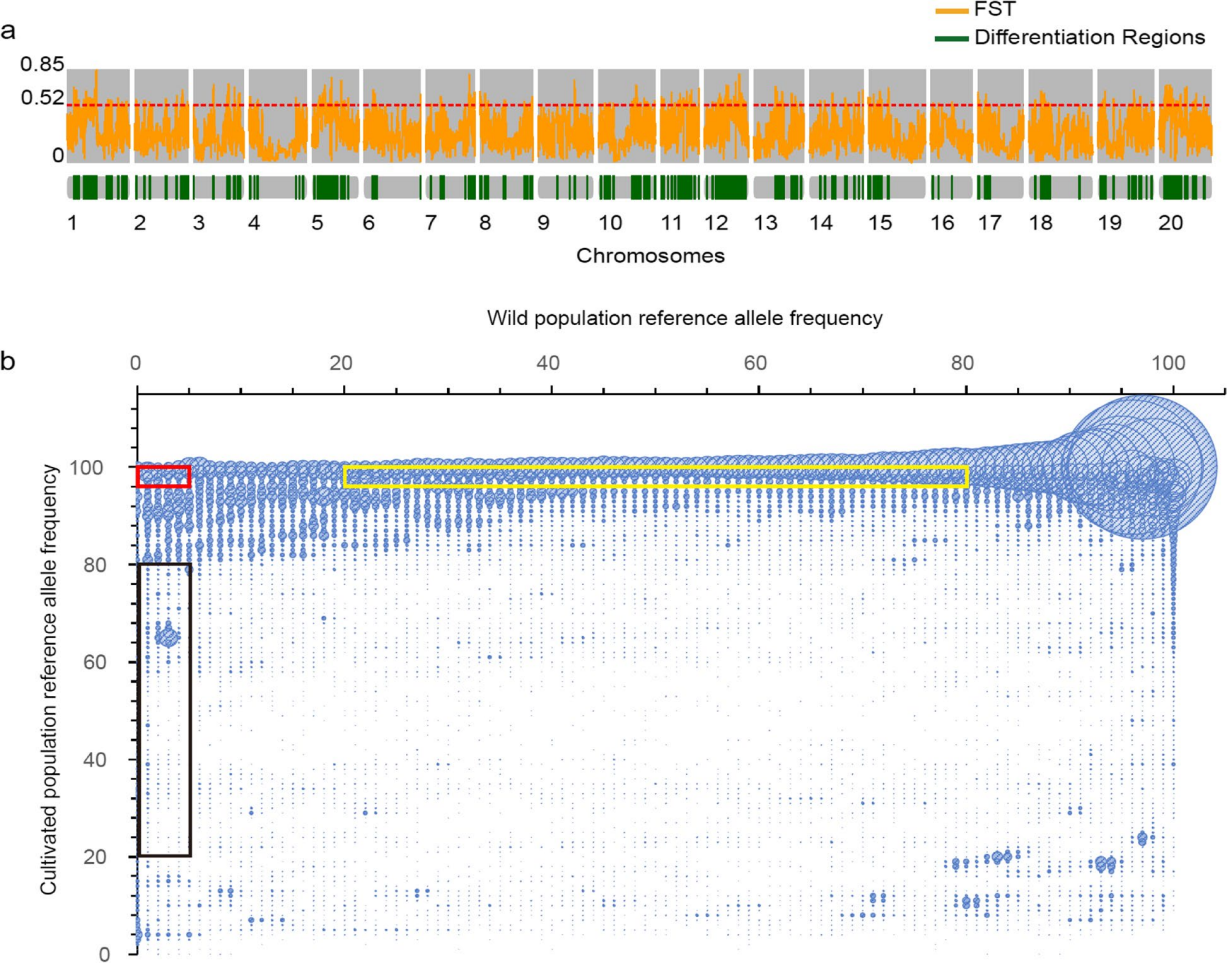
Differentiation regions between wild and cultivated soybean populations and the SNP allele frequency in the differentiation regions. **a** The distribution of fixation index (Fst) and differentiation regions along the chromosomes. Yellow lines represent Fst values, and Fst values above the red dashed line represent the top 5% Fst values of the genome. The green bars indicate the genomic differentiation regions between the wild and cultivated soybean populations. **b** Bubble chart of SNP allele frequency in the differentiation regions. The ordinate represents the frequency of reference alleles in the wild population, the abscissa represents the frequency of reference alleles in the cultivated soybean population, and the circle size represents the number of SNPs at that frequency. The red, black, and yellow boxes represent the allele frequency distributions of type 1, type 2, and type 3 SNPs, respectively

A total of 10,597,683 SNPs from 418 domesticated (*Glycine max*) and 345 wild (*Glycine soja*) soybean accessions published in a previous study [13] were used as the test population for identifying the genetic differentiation regions between wild and cultivated soybean populations. In all, 324 genomic regions were identified (fixation index [Fst] > 0.52, top 5%), and their distribution on the chromosomes was shown in Fig. 1a. Within these regions, 710,631 SNPs were identified, with 24,493 of which being exonic (including 13,866 nonsynonymous SNPs). Due to the reference genome having been constructed with the cultivated soybean Williams 82, the cultivated populations exhibited high reference genome allele frequencies. However, many SNPs in wild populations also showed high reference genome allele frequencies, implying that these SNPs in the differentiation regions are rare SNPs with small minor allele frequencies (Fig. 1b). In previous studies, 5% was considered as the dividing line between low-frequency and high-frequency alleles [17], so we used 5% as the threshold for SNPs that are close to fixed in the population. For SNPs with good polymorphism in the population, allele frequencies between 0.2 and 0.8 are a rational trade-off between precision and sensitivity [18]. Therefore, we set the reference allele frequency at 20% to 80% to ensure good polymorphism of the marker in the population. Hence, the representative SNPs were further sorted into the following types (Fig. 1b). Type 1 SNPs were those with allele frequencies close to fixed but still different between wild and cultivated soybean populations, with cultivated dominant allele frequencies (reference genome allele frequencies) of less than 5% in the wild populations and greater than 95% in the cultivated populations. For type 2 SNPs, the cultivated dominant allele frequencies in wild soybeans had to be less than 5%, and between 20–80% for the cultivated populations. Type 3 SNPs were those with cultivated dominant allele frequencies between 20–80% in the wild populations and greater than 95% in the cultivated populations.

**Fig. 2:**
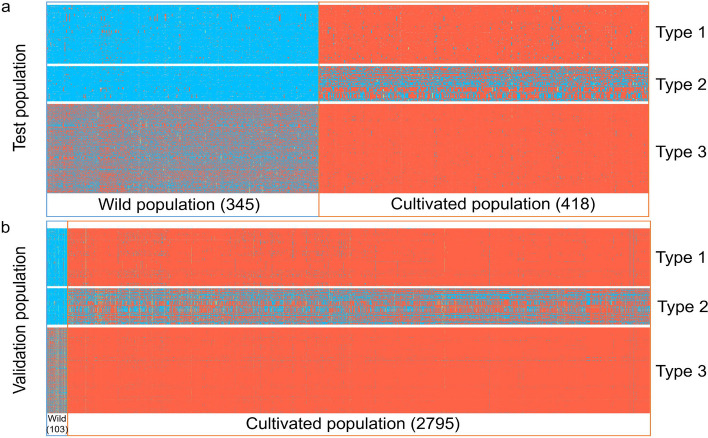
The genotypes of candidate exonic SNPs in the test and validation populations of soybean.** a** The genotypes of candidate exonic SNPs in the test population. **b** The genotypes of candidate exonic SNPs in the validation population. Orange blocks represent reference genotypes; blue blocks represent alternative genotypes; green blocks represent heterozygous genotypes; and gray blocks represent missing genotypes

For type 1, type 2 and type 3 SNP sets, 8,207, 4,422 and 156,464 SNPs were identified, among which 343, 134 and 5,176 were exonic SNPs, respectively. This result reflects a higher genetic diversity among the wild populations than within the cultivated ones, which is in line with previous studies [19]. The genotype heatmap showed that the three types of SNP sets followed distinct patterns between wild and cultivated populations (Fig. 2a).

### Gene functions of the differentiation regions between wild and cultivated soybeans

Generally speaking, the genotypes of the type 3 SNP-related genes were nearly fixed in cultivated populations but still had the highest number of polymorphisms in wild populations among the genes associated with the three types of candidate SNP markers. These genes were enriched in 55 gene ontology (GO) terms, with the top 5 being ATPase activity, telomere maintenance, DNA recombination, DNA helicase activity, and DNA repair (Tables S2 and S3). The results indicated that the genomes of wild soybeans had higher genomic instability, therefore more readily adaptable to changing environments. The elimination of genomic instability in domesticated varieties was associated with the domestication process [20]. In addition, some agronomic traits-related genes of soybean were identified as type 3 SNP-related genes, such as *GmCYP78A72* (*Glyma.19G240800*) which is related to soybean seed size [21], *GmLEC2a* (*Glyma.20G035800*) which is involved in controlling the biosynthesis and catabolism of seed storage substances and seed development [22] and *GmZF392* (*Glyma.12G205700*) which controls lipid accumulation in soybeans [23].

The type 1 SNP-associated genes, nearly fixed in both wild and cultivated soybean populations but differentiated between them, were enriched in 19 GO terms (*P* < 0.05), including ATPase activity and protein refolding, which play an important role in plant growth and development [24, 25]. For example, *Glyma.13G241700* was predicted to encode a transmembrane transporter-like protein for the biosynthesis of the bloom in the pod endocarp. The mutation of this gene in cultivated soybeans results in a shiny seed surface in domesticated soybeans [26]. These genes were speculated to be related to the predominant differential phenotypes between wild and cultivated soybeans (Tables S4 and S5).

The type 2 SNP-related genes had genotypes that are nearly fixed in the wild populations but still had a degree of polymorphism in the cultivated populations. This group of genes have often been overlooked in previous domestication studies due to their contradiction of the theoretical assumption of reduced polymorphism from wild to cultivated populations. There were fewer genes in this category than the type 1 and type 3 SNP-related genes, and were enriched in nine GO terms, including the integral component of membrane, and tRNA methyltransferase activity related to gene transcription and metabolic activity. These may be related to agronomic traits resulting from the domestication process (Tables S6, S7). For example, *Glyma.17G128200* and *Glyma.10G295400* are associated with soybean seed yield traits [27, 28], *Glyma.15G130000* is associated with soybean seed protein concentration [29], *Glyma.11G092100* was demonstrated to directly influence the anabolism of sucrose in soybean [30], and *Glyma.12G143200* and *Glyma.13G285300* are associated with soybean cyst nematode and root-lesion nematode resistance, respectively [31, 32].

### Validation of the candidate SNPs using a different soybean population

The validation population included 2,795 domesticated (*G. max*) and 103 wild (*G. soja*) soybean accessions published in another study [12]. We conducted quality control of the samples from the test and validation populations, and the results showed that only 12 samples were duplicated between the two populations (Table S1). We mapped the candidate SNPs with their flanking 100-bp sequences to the ZH13 v2 genome [33] with no InDels allowed within the flanking sequences. The results showed that 98.60% of the candidate exonic SNPs could be found in the SNP dataset of the validation population [12], demonstrating that these SNPs are highly conserved in both the wild and cultivated soybean populations. The result also demonstrated the feasibility of SNP dataset comparisons across different reference genomes. The genotype heatmap of the SNPs indicated that the genotype patterns of the validation population were similar to those in the test population, indicating that the genotypes of these SNPs were also differentiated between wild and cultivated soybean populations in the validation population (Fig. 2b).

### Evaluation of the test and validation population accessions

**Fig. 3:**
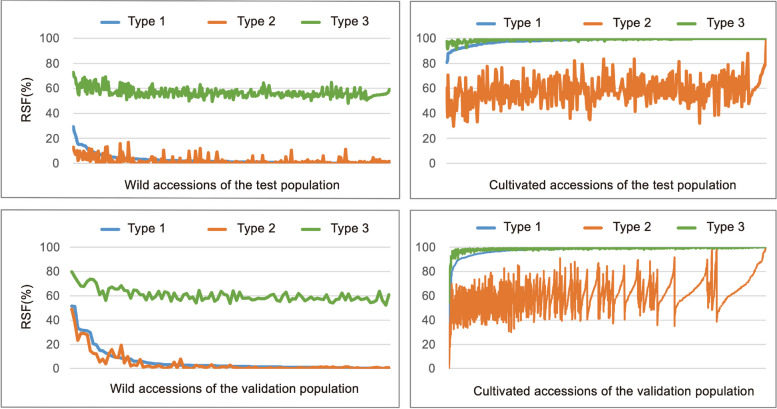
The RSF values of each accession in the test and validation populations. Blue lines represent the RSF of type 1 SNPs. Red lines represent the RSF of type 2 SNPs. Green lines represent the RSF of type 3 SNPs

To facilitate the evaluation of each accession, we calculated the homozygous SNP frequencies of cultivated soybean types (reference SNP frequency [RSF]) in each accession. In the test population, for type 1 SNPs, all wild soybeans had an RSF less than 29.33% while all cultivated soybeans had an RSF greater than 80.70%. For type 2 SNPs, all wild soybeans had an RSF less than 16.79%, while all cultivated soybeans had an RSF greater than 91.64% for type 3 SNPs (Table 1, Table S8, Fig. 3). These results showed that RSF can be used to effectively distinguish between wild and cultivated soybeans in the test population.

**Table 1:** The range of reference SNP frequency (RSF) values of wild versus cultivated soybean accessions

SNP type	RSF range of wild soybeans	RSF range of cultivated soybeans
Type 1	0–29.33%	80.70–100%
Type 2	0–16.79%	29.85–99.16%
Type 3	48.26–72.87%	91.64–100%

In the validation population, for type 1 SNPs, 94.17% (97/103) of wild soybeans had an RSF < 30% and 99.11% (2770/2795) of cultivated soybeans had an RSF > 80% (Table S9, Fig. 3). When the six wild soybeans with an RSF > 30% for type 1 SNPs were further analyzed, it was found that more than 50% of these SNPs were heterozygous in three of the accessions (s11, s60 and s897), indicating that these three samples may be hybrids. For the other three accessions (s48, s891 and s892), we speculated that they are highly likely cultivated soybeans. Of the 25 cultivated samples with an RSF < 80% for type 1 SNPs, three had an RSF < 30% (s2822, s2804, s2130), indicating that they are highly likely to be wild soybeans. To verify the wild/cultivated nature of these anomalies, a neighbor-joining (NJ) tree was then constructed using all the genome-wide SNPs. The results showed that the three cultivated soybeans (s2822, s2804 and s2130) were in the clade of the wild soybean population, while the three wild soybeans (s48, s891 and s892) were in the clades of cultivated soybeans (Fig. S2). The result of the NJ tree is consistent with our hypothesis that only a small number of type 1 SNPs are needed to accurately distinguish between cultivated and wild soybeans. For the other 22 samples of cultivated soybean with an RSF between 30 and 80%, seven are improved cultivars, and 15 are landraces distributed across the cultivated branches. One accession (s150) had an RSF of 76.63%. According to the information provided [12], it is a hybrid of Hu Pi Dou (*G. max*) and Ye Sheng Dou J15 (*G. soja*). For other accessions, we could not accurately indicate the genetic background of these samples due to the lack of sample information in the source study. Nonetheless, based on the above results, the following criteria can be reasonably adopted to distinguish between wild and cultivated soybeans using type 1 SNPs. If the RSF value is less than 30% for a soybean sample, it is most likely a wild soybean. If the RSF value is between 30–80%, it could be a wild, cultivated or hybrid soybean. If the RSF value exceeds 80%, it is most likely a cultivated soybean. However, considering that all the wild soybeans in both the test and validation populations had RSFs below 50% for type 1 SNPs, and all the cultivated soybeans in both populations had RSFs greater than 50% for these SNPs, 50% can be used conveniently as a threshold for separating cultivated soybeans from wild ones.

### Trimming the SNP sets to accommodate low-density SNP array platforms

To reduce the cost of effectively evaluating wild and cultivated soybeans, we trimmed the SNP sets based on physical distance (set at a minimum of 20 Mb between SNPs), reducing the total to 93 markers (with 31, 22 and 40 for type 1, type 2 and type 3 SNP markers, respectively). The genotypes of these SNPs in the test and validation populations are shown in Fig. S3, and the results show that the genotype patterns and the RSF distribution maps in wild and cultivated populations resembled the pre-trimming SNP sets. We analyzed the RSF of each wild accession in the validation population and found that nine samples had an RSF greater than 20%, consistent with the results before trimming. For the cultivated accessions in the validation population, 97.85% of the samples had an RSF greater than 80%, which was slightly lower than the 99.11% before trimming (Fig. S4, Tables S10 and S11). These results showed that even if the total number of SNP markers is reduced to below 100, we can still get a reliable judgment due to the stable population allele frequency. These high-quality SNPs can therefore be used to design low-density SNP arrays for platforms such as Kompetitive Allele Specific PCR (KASP) [34] and Genotyping by Pinpoint Sequencing of multiplex PCR products (mGPS) [35].

### Using amplification refractory mutation system–polymerase chain reaction (ARMS-PCR)-based SNPs to distinguish between wild and cultivated soybeans

**Fig. 4:**
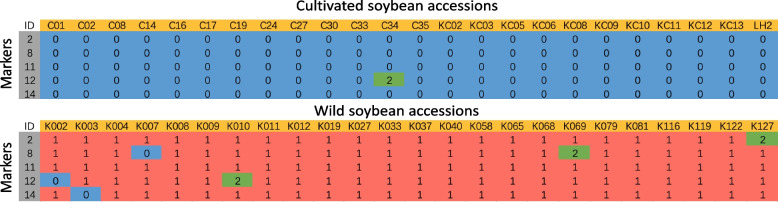
The genotypes of 48 soybean samples were tested using five SNP markers. Blue blocks (0) represent the reference genotype; red blocks (1) represent the alternative genotype; green blocks (2) represent the heterozygous genotype

PCR-based testing using a small number of markers is convenient and efficient. The ARMS-PCR-based SNPs determine whether a soybean sample is wild or cultivated by statistically testing the probability of each SNP marker occurring within the sample against a binomial distribution. For type 1 SNPs, the probability of each SNP is greater than 95%, and an RSF of 50% can be used as the minimum threshold criterion for the evaluation. Using just five ARMS-PCR-based markers in the evaluation panel, a greater-than-99.88% judgement accuracy rate can be achieved (Table S12). For the PCR experiment, 17 type 1 SNP markers located on different chromosomes were selected for the test panel (Table S13). A cultivated soybean (C14) and a wild soybean (K122) with 20X Illumina sequencing data were used to test these markers. Sequencing data showed that these 17 SNPs had different genotyping patterns in the two genomes and therefore could be used to test the marker validity. After PCR testing, five best-quality markers were chosen for further analyses (Fig. S5 ). These five markers were then used to genotype 24 cultivated and 24 wild soybeans (Figs. S6-S10). The results showed that these 48 samples could be effectively identified as either wild or cultivated using only five SNP markers (Fig. 4).

### Development of InDel markers

In addition, InDel markers were also selected using the same process as for SNP markers. For type 1, type 2, and type 3 InDel data sets, 575, 451, and 17,992 InDels were identified, with 14, 3, and 105 of which being exonic, respectively. In total, these InDels involved 107 genes (Table S14). Among the exonic InDels, 92.62% were validated in the validation population, demonstrating the conserved genotype frequencies of these markers in both wild and cultivated populations. The selected InDels are therefore as reliable for subsequent breeding and germplasm resource studies as the SNP markers.

The genes affected by type 1 InDels were nearly fixed in either the wild or the cultivated soybean populations. The gene list showed that these genes were involved in many physiological functions in cell differentiation, plant growth and development (Table S15). Examples of such genes include those encoding plasmodesmata-located protein 8 (*PDLP8*) [36], multidrug and toxic compound extrusion (*MATE*) proteins [37], CINCINNATA (CIN)-like TEOSINTE BRANCHED 1/CYCLOIDEA/PCF (*TCP*) family transcription factor 4 [38], among others. Among the proteins encoded by the three type 2 InDel-affected genes, hydroxyproline-rich glycoproteins (*HRGPs*) are a superfamily of plant cell wall proteins that function in diverse aspects of plant growth and development [39], and *MYB86* is involved in negative regulation and plays an important role in plant resistance to abiotic stress [40]. The products of genes affected by type 3 InDels are associated with soybean domestication. These include *PIF1* helicases, disease resistance family protein / LRR family protein, Cytochrome P450 superfamily proteins, NAD(P)H dehydrogenase B2, and others. All these proteins are major regulators and contributors to plant growth and development [41] (Table S15).

### Genes with large-effect mutation (LEM) in the population and their characteristics

In resequencing studies, rare SNPs with low minor allele frequency (MAF) values were usually discarded to avoid sequencing errors. Based on the SNP and InDel datasets in the test and validation populations, more than 80% of the SNPs and InDels had MAFs less than 0.01 in the validation population, and more than 75% of the InDels had MAFs less than 0.01 in the test population (Table S16). In both populations, mutated genes with high-frequency LEM (MAF ≥ 0.01) accounted for 15.09% of the total gene count in the test population and 23.56% of the total gene count in the validation population (Table 2).

**Table 2:** Genes with large-effect mutations in test and validation populations

Population	Mutation type	All genes	Mutated genes	MAF < 0.01	MAF ≥ 0.01
Validation	SNP	59,313^a^	17,323	15,280	4,067
	InDel		27,591	25,557	6,333
	SNP & InDel		34,058 (57.42%)	31,902 (53.79%)	8,948 (15.09%)
Test	SNP	56,044^b^	5,175	NA	5,175
	InDel		29,434 (52.52%)	26,540 (47.36%)	10,430
	SNP & InDel		30,449 (54.33%)	26,540	13,203 (23.56%)

*MAF* minor allele frequency

^a^gene set of ZH13 v2

^b^gene set of Willams 82 v2

Genes in the validation population were classified into two categories: genes without LEMs and genes affected by high-frequency LEMs (MAF ≥ 0.01). GO and Kyoto Encyclopedia of Genes and Genomes (KEGG) analyses indicated that genes without LEMs were mainly enriched in basic biological functions. For example, the most significant GO term was "structural constituent of ribosome," and the most significant KEGG pathway was "photosynthesis" (Table S17, Fig. S11). The genes with high-frequency LEMs did not show GO enrichment, KEGG analyses indicated their association with environmental adaptations, such as "plant-pathogen interaction," "glyoxylate and dicarboxylate metabolism," and "ABC transporters" (Fig. S12).

### Construction of a natural mutant database

To eliminate the influence of sequencing and genotyping errors, we superimposed the high-frequency (MAF ≥ 0.01) LEMs of the test and validation populations. There were 6,964 high-frequency large-effect SNPs and 20,565 large-effect InDels in the test population, among which 5,721 SNPs and 12,069 InDels overlapped with those in the validation population. However, when comparing the functional annotations of the LEMs between the two populations, we found that only 52.94% of the large-effect SNPs and InDels had the same annotation (Table S18). This indicated that different reference sequences could significantly affect the annotation results of the variations in soybean resequencing studies, possibly since soybean is a paleotetraploid, which affects the accuracy of gene annotation. To minimize the impact of different reference sequences, we compiled a natural mutant database by selecting 5,755 genes (including 9,418 LEMs) that are shared between the two populations and have the same variant annotation information, including gene names, mutation types, and the detailed accessions. The natural mutant database was uploaded to Figshare under the link: https://doi.org/10.6084/m9.figshare.24912585 and included in Additional file 1–2.

## Discussion

In this study, the resequencing data from two populations were used to screen for SNPs and InDels between and within wild and cultivated soybean populations. Compared to previous studies [6–11], the accuracy and effectiveness of the thus-selected markers were ensured by cross-validation between the test and validation populations. Additionally, the differentiation between wild and cultivated populations, as well as the polymorphism within both wild and cultivated populations, were taken into consideration. However, this study only focused on the variations in coding regions, whereas the importance of non-coding region variations is gradually gaining attention and deserves further in-depth research [42].

Our results showed that just five ARMS-PCR-based SNP markers are needed to distinguish between wild and cultivated soybeans. Even the identities of previously misclassified wild and cultivated samples from the validation population [12] were resolved using our markers. For example, three cultivated soybeans (s2804, s2812, and s2130), according to the phylogenetic tree, should be classified as wild samples. These three cultivated soybeans were also assigned to the wild clade in another study [16], supporting our observation here. Using a small set of ARMS-RCR-based SNP markers is very convenient and economical for identifying germplasm resources.

Furthermore, SNP markers were selected in this study to not only differentiate between wild and cultivated soybean populations, but to also represent polymorphisms within wild or cultivated populations. For the latter purpose, these markers were classified into three types. Type 3 markers exhibit a high degree of polymorphisms in wild populations and a low degree of polymorphisms in cultivated populations, representing those genes under selection during soybean domestication. Type 1 markers represent differentiated and dominant genes in both wild and cultivated populations. Type 2 markers reflect polymorphisms resulting from soybean domestication. The type 1 and 2 markers have received less attention in previous population studies [13, 43, 44].

Exploring naturally mutated genes, especially those occurring at low frequency, is crucial in crop improvement. However, due to their rarity, they can only be discovered through large populations, and careful validation is also required to eliminate genotyping errors. Here, it was found that genes with no LEMs are mostly associated with fundamental biological functions, while genes experiencing LEM mutations in the population are often related to environmental adaptations. The natural mutant library constructed in this study utilized the resequencing data of 3,661 soybean accessions. It underwent cross-validation in the test and validation populations to ensure accuracy, and the gene set thus identified through screening with LEMs will be valuable for soybean breeding and reverse genetics research. Since these mutations were accumulated by naturally occurring soybean accessions, their utilization in soybean breeding will contribute to addressing food security without raising ethical concerns commonly associated with genetically modified organisms (GMOs) utilizing genes from a different species. While constructing the mutant library, we discovered the significant impact of reference sequences on mutation detection and functional studies. With the advancement of sequencing technologies, it is believed that higher-quality genomes and more accurate gene annotation information will help address the challenges in this aspect [33, 45].

## Conclusion

This study provides insight into the effective utilization of resequencing data from 3,661 soybean accessions for selecting useful DNA markers. The efficient markers obtained here can be applied in germplasm identification, soybean domestication, fingerprinting, and soybean breeding research. Additionally, the natural mutant library constructed in this study offers a convenient tool for soybean breeding and reverse genetics research.

## Methods

### Data collection of the test and validation population

The test population includes 418 domesticated (*Glycine max*), 345 wild (*Glycine soja*), and 18 natural hybrid (*G. max*/*G. soja*) accessions [13]. Its genome-wide variation map contains 10.6 million SNPs and 1.4 million InDels [13]. The validation population includes 103 wild (*G. soja*), 1,048 landraces (*G. max*), and 1,747 cultivars (*G. max*) soybean accessions [12]. Its genome-wide variation map contains 31.9 million SNPs and 6.1 million InDels [12].

### Identification of differentiation genetic regions between wild and cultivated soybeans

The fixation index (Fst) between wild and cultivated soybean populations was calculated for each 100-kb sliding window with a step size of 10 kb using VCFtools v0.1.13 [46]. The windows that contained less than 10 SNPs were excluded from further analyses, and the top 5% of windows were considered differentiation regions.

### Gene-based SNP and InDel annotations

The ANNOVAR (v2013–06–21) package [47] was used for gene-based SNP and InDel annotations according to the gene annotation of the WM82v2 genome (http://ftp.ensemblgenomes.org/pub/plants/current/gff3/glycine_max/Glycine_max.Glycine_max_v2.1.54.gff3.gz).

### Functional annotation of gene lists

Geno ontology (GO) annotation of gene lists was performed using DAVID [48] with Ensembl gene ID as the identifier and *Glycine max* as the background.

### Selection of candidate SNPs

While assessing the allele frequencies of SNPs in the differentiation region, most of the reference allele frequencies were found to be greater than 80% in the cultivated population because the reference sequence used was for the cultivated soybean Williams 82. To narrow down the search for the candidate representative SNPs, the SNPs in the differentiation region were classified into the following three categories (Fig. 2):

Type 1: SNPs that are nearly fixed in both wild and cultivated soybeans, with the reference allele frequency screening criterion set at less than 5% in the wild population and greater than 95% in the cultivated population.

Type 2: SNPs that are nearly fixed in cultivated soybeans but have good polymorphism in wild soybeans, with the reference allele frequency screening criterion set at less than 80% but greater than 20% in the wild population and greater than 95% in the cultivated population.

Type 3: SNPs that are nearly fixed in wild soybeans but have good polymorphism in cultivated soybeans, with a reference allele frequency screening criterion set at less than 5% in the wild population and less than 80% but greater than 20% in the cultivated population.

### Mapping SNPs and InDels across different reference genomes

To unify the genotyping information based on different reference genomes used in multiple population studies, the following steps were taken:

- 1) One hundred-base pair flanking sequences for each SNP were extracted to generate a 201-bp SNP sequences file (Fasta format).
- 2) Using the MEM algorithm of the Burrow-Wheeler Aligner (BWA, Version: 0.7.17-r1188) software [49], the SNP sequences were then mapped to the reference genome for comparison.
- 3) The best hit was selected, with no InDels in the flanking sequences.
- 4) Position transformation and further confirmation of allele consistency were performed.

### Samples and DNA extraction

Twelve wild and twelve cultivated soybean accessions were grown by germinating their seeds on 0.8% water agar in sterile magenta boxes at 28°C in the dark. After 3-4 days, the hypocotyls and radicals were harvested from the young seedlings and immediately frozen in liquid nitrogen. DNA was extracted from the frozen hypocotyls and radicals using the DNeasy Plant Mini Kit (Qiagen, Hilden, Germany, Cat# 69104). The source of the soybean seeds is listed in Table S19.

### SNP genotyping by ARMS-PCR method

The tetra-primer amplification refractory mutation system–polymerase chain reaction (ARMS-PCR) method was adopted for quick genotyping with a small SNP set [50].

SNP selection criteria for ARMS-PCR:

- 1. No InDels within 500 bp on both sides of the SNP.
- 2. Select one marker for each chromosome.
- 3. Pass the primer design.

Primers were designed using the program developed by Ye (http://primer1.soton.ac.uk/primer1.html) [51] with the following changes to the parameters:

- a. The maximum relative size difference between the two inner amplicons is 2.0.
- b. The minimum relative size difference between the two inner amplicons is 1.2.
- c. The maximum (inner) amplicon size is 500.
- d. The minimum (inner) amplicon size is 200.
- e. The optimum (inner) amplicon size is 250.

The primer sequences for testing the SNPs are listed in Table S13, and the PCR reagents and steps are listed in Tables S20 and S21. The PCR products were visualized in 2% agarose gel stained with ethidium bromide.

### Supplementary Information

Supplementary Material 1.

Supplementary Material 2.

Supplementary Material 3.

Supplementary Material 4.

## Data Availability

The sequencing data for this project have been deposited in the NCBI Sequence Read Archive (SRA) (http://www.ncbi.nlm.nih.gov/sra) under accession number PRJNA1062436. The natural mutant database was uploaded to Figshare (https://doi.org/10.6084/m9.figshare.24912585) and included in Additional file 1–2. All other data supporting this research have been included in this article and the supplementary information.

## Abbreviations

SNP: Single nucleotide polymorphism
InDel: Insertion/Deletion
MAF: Minor allele frequency
RSF: Reference SNP frequency
LEM: Large-effect mutations
GO: Gene ontology
KEGG: Kyoto Encyclopedia of Genes and Genomes
Fst: Fixation index
