# Supplementary material for: Developing an SNP dataset for efficiently evaluating soybean germplasm resources using the genome sequencing data of 3,661 soybean accessions
Source: BMC Genomics. 2024 May 14;25:475. doi: 10.1186/s12864-024-10382-3 (PMC11092025; doi:10.1186/s12864-024-10382-3)
Supplement: Supplementary file 3 — Supplementary Material 3. [file 12864_2024_10382_MOESM3_ESM.docx]

**Additional files**

**Additional file 1**. Large-effect SNPs mutant genes library.csv. This file includes the large-effect SNPs across the test and validation populations, including the genome location information on the ZH13 v2 assembly and Willams 82 v2 assembly, gene annotation information, and genotype information for all accessions.

**Additional file 2**. Large-effect InDels mutant genes library.csv. This file includes the large-effect InDels across the test and validation populations, including the genome location information on the ZH13 v2 assembly and Willams 82 v2 assembly, gene annotation information, and genotype information for all accessions.
