# Supplementary material for: Developing an SNP dataset for efficiently evaluating soybean germplasm resources using the genome sequencing data of 3,661 soybean accessions
Source: BMC Genomics. 2024 May 14;25:475. doi: 10.1186/s12864-024-10382-3 (PMC11092025; doi:10.1186/s12864-024-10382-3)
Supplement: Supplementary file 4 — Supplementary Material 4. [file 12864_2024_10382_MOESM4_ESM.pdf]

Supplementary figures

Figure S1 A schematic workflow of this study and the potential uses of the SNP dataset generated.

Figure S2 A neighbor-joining (NJ) tree of 2,898 soybean accessions from Liu's study. The tree was constructed using the program TreeBeST (v1.92) with the whole genome SNP data and displayed using Itol. Red branches represent wild soybeans; green branches represent landraces; orange branches represent improved soybean cultivars.

Figure S3 The genotype heat map of the thinned exonic SNPs of the test and validation populations. Orange blocks represent the reference genotype; blue blocks represent the alternative genotype; green blocks represent the heterozygous genotype; and gray blocks represent a missing genotype.

Figure S4 The RSF values of each accession in the test and validation populations using the thinned SNP set. Blue lines represent the RSF of type 1 SNPs. Red lines represent the RSF of type 2 SNPs. Green lines represent the RSF of type 3 SNPs

Figure S5 The gel pictures of the 17 SNPs tested with K122 and C14. High-quality SNPs are highlighted with green rectangles.

Figure S6 Agarose gel images of the results of SNP 2 testing on 48 soybean germplasms by PCR. a Twenty-four wild and cultivated germplasms. **b**. Twenty-four wild and cultivated germplasms. Accessions with different genotypes in the population were highlighted in red.

Figure S7 Agarose gel images of the results of SNP 8 testing on 48 soybean germplasms by PCR. a Twenty-four wild and cultivated germplasms. **b** Twenty-four wild and cultivated germplasms. Accessions with different genotypes in the population were highlighted in red.

Figure S8 Agarose gel images of the results of SNP 11 testing on 48 soybean germplasms by PCR. a Twenty-four wild and cultivated germplasms. **b** Twenty-four wild and cultivated germplasms.

Figure S9 Agarose gel images of the results of SNP 12 testing on 48 soybean germplasms. a Twenty-four wild and cultivated germplasms. **b** Twenty-four wild and cultivated germplasms. Accessions with different genotypes in the population were highlighted in red.

Figure S10 Agarose gel images of the results of SNP 14 testing on 48 soybean germplasms. a Twenty-four wild and cultivated germplasms. **b** Twenty-four wild and cultivated germplasms. Accessions with different genotypes in the population were highlighted in red.

Figure S11 Enriched KEGG pathways of non-LEM genes.

Figure S12 Enriched KEGG pathways of LEM genes with MAF ≥ 0.01.

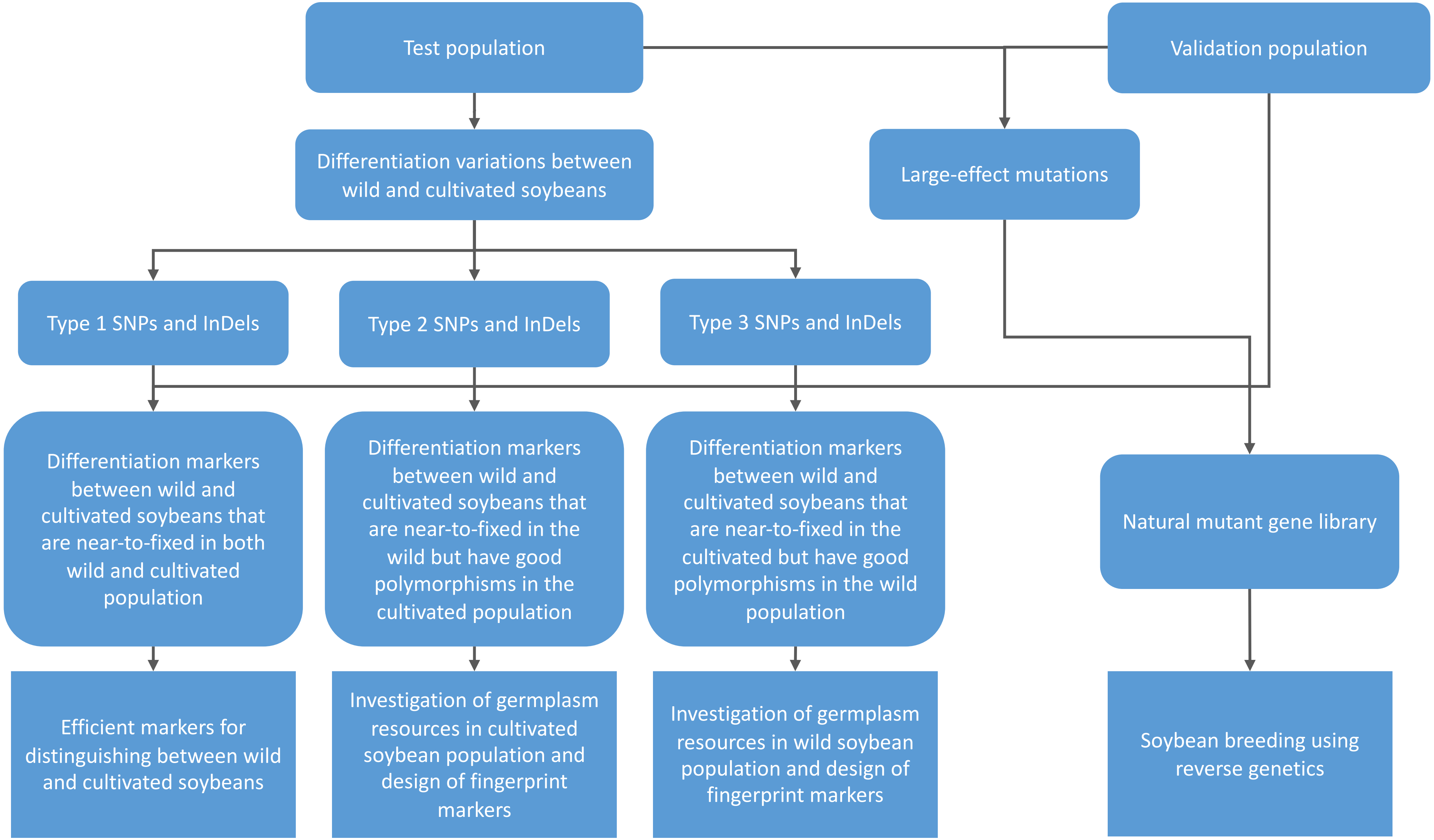

Figure S1 A schematic workflow of this study and the potential uses of the SNP dataset generated.

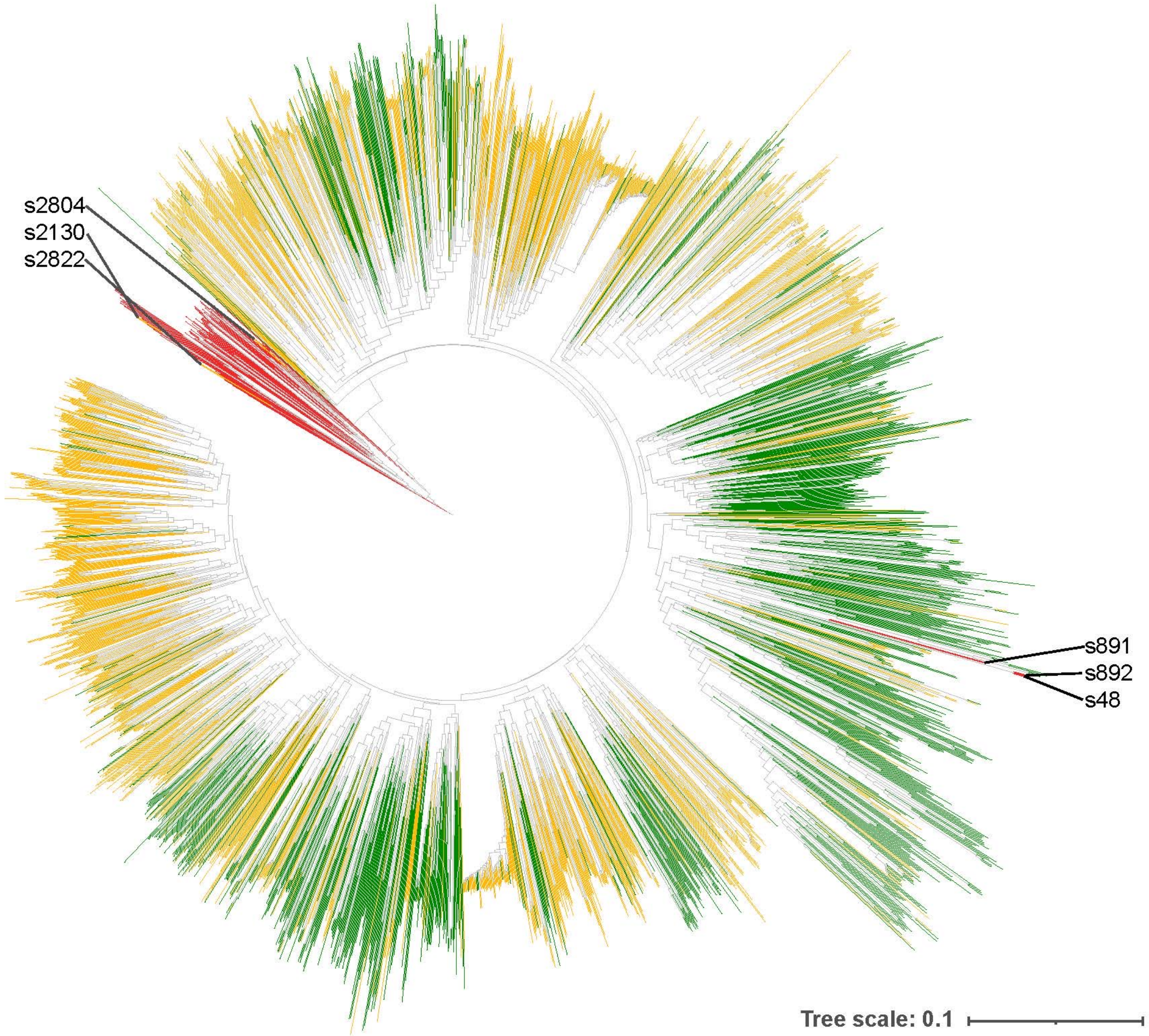

Figure S2 A neighbor-joining (NJ) tree of 2,898 soybean accessions from Liu's study. The tree was constructed using the program TreeBeST (v1.92) with the whole genome SNP data and displayed using Itol. Red branches represent wild soybeans; green branches represent landraces; orange branches represent improved soybean cultivars.

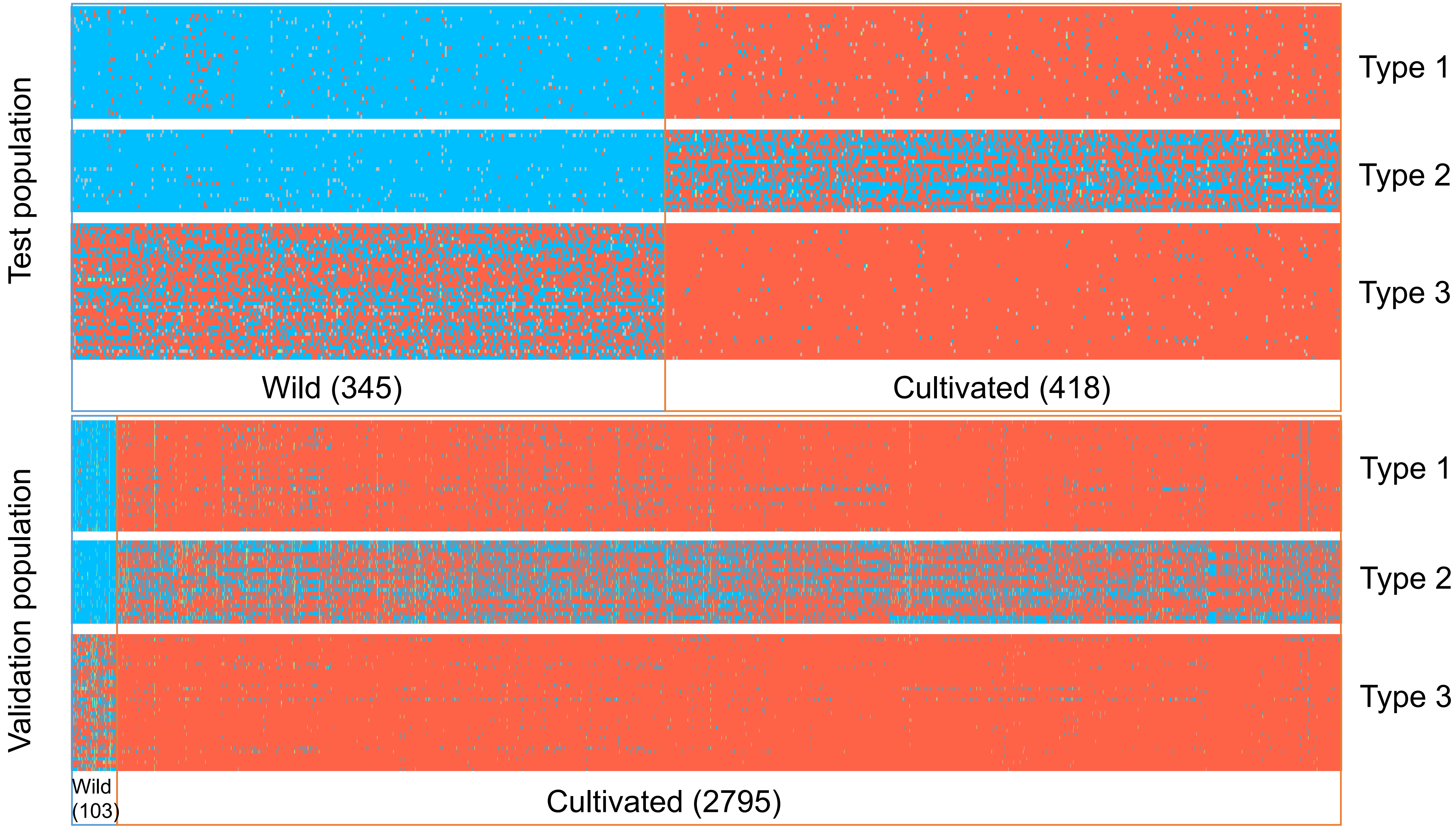

Figure S3 The genotype heat map of the thinned exonic SNPs of the test and validation populations. Orange blocks represent the reference genotype; blue blocks represent the alternative genotype; green blocks represent the heterozygous genotype; and gray blocks represent a missing genotype.

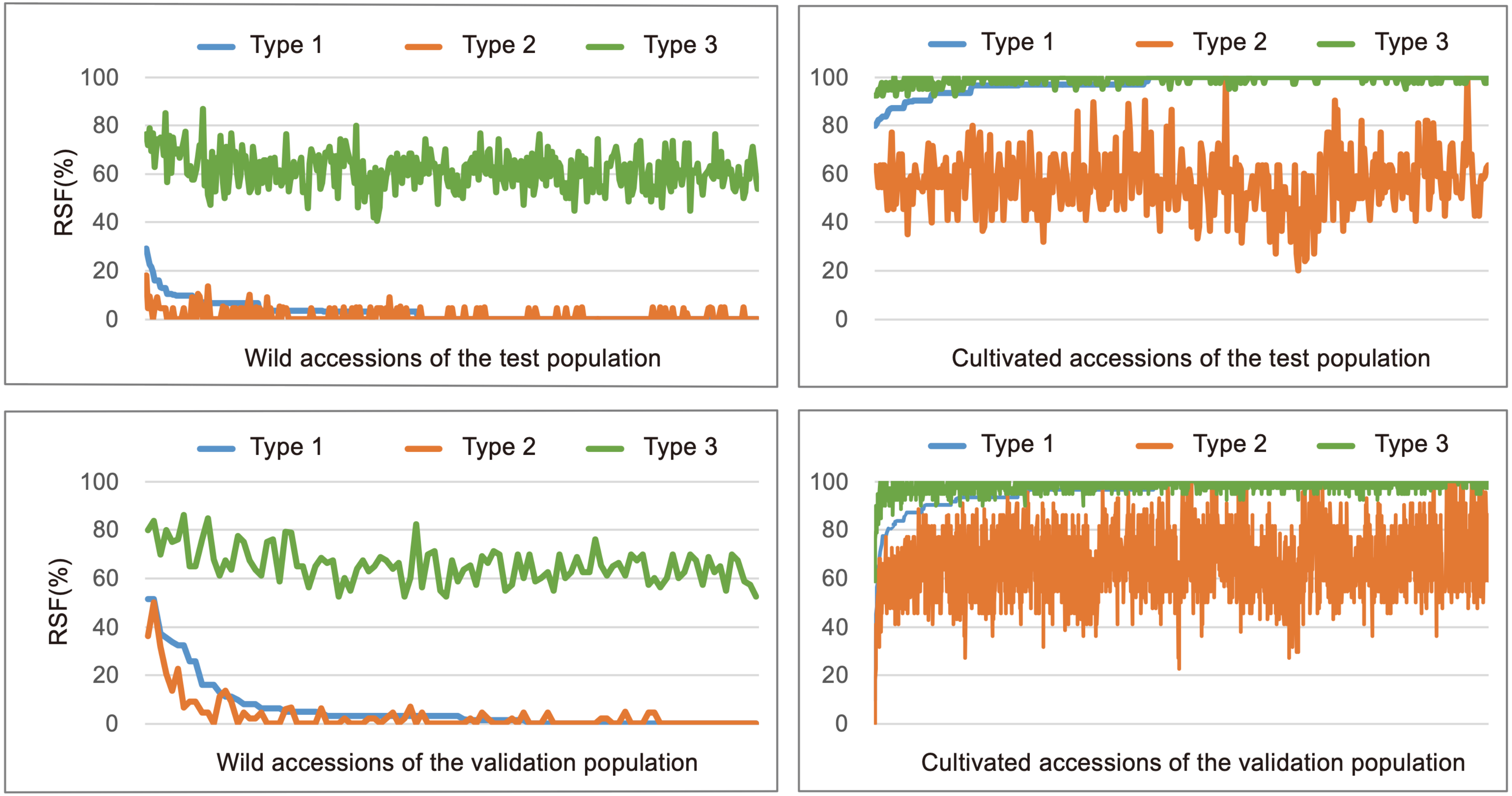

Figure S4 The RSF values of each accession in the test and validation populations using the thinned SNP set. Blue lines represent the RSF of type 1 SNPs. Red lines represent the RSF of type 2 SNPs. Green lines represent the RSF of type 3 SNPs

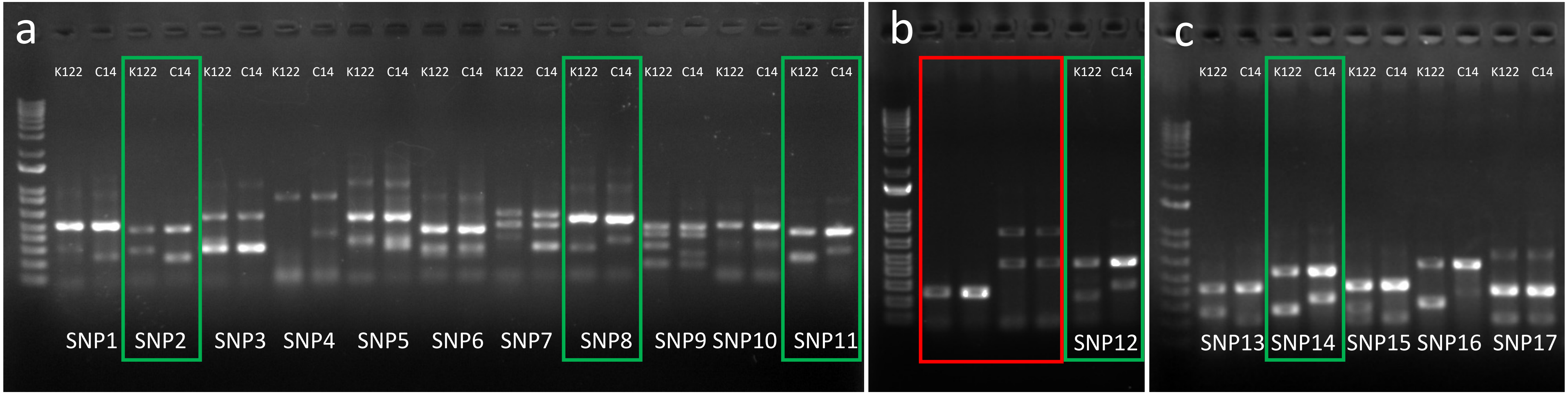

Figure S5 The gel pictures of the 17 SNPs tested with K122 and C14. a The gel picture of SNP 1-11. **b** The gel picture of SNP 12. **c** The gel picture of SNP 13-17. High-quality SNPs are highlighted with green rectangles. Markers in the red rectangle are not relevant to this study.

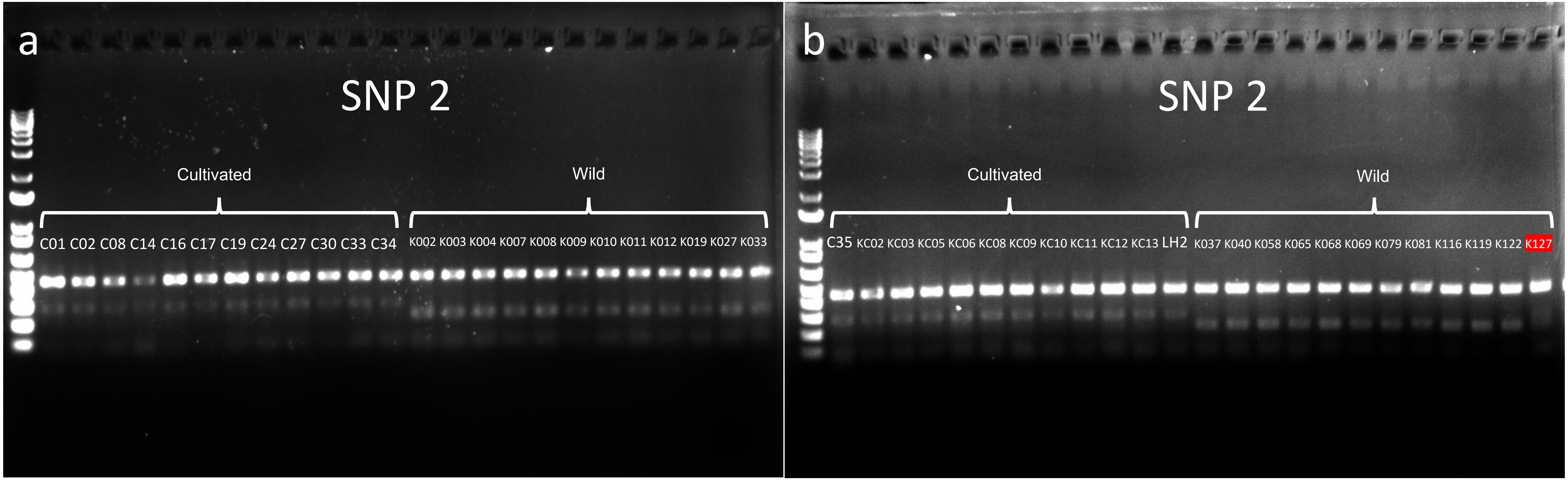

Figure S6 Agarose gel images of the results of SNP 2 testing on 48 soybean germplasms by PCR. a Twenty-four wild and cultivated germplasms. **b** Twenty-four wild and cultivated germplasms. Accessions with different genotypes in the population were highlighted in red.

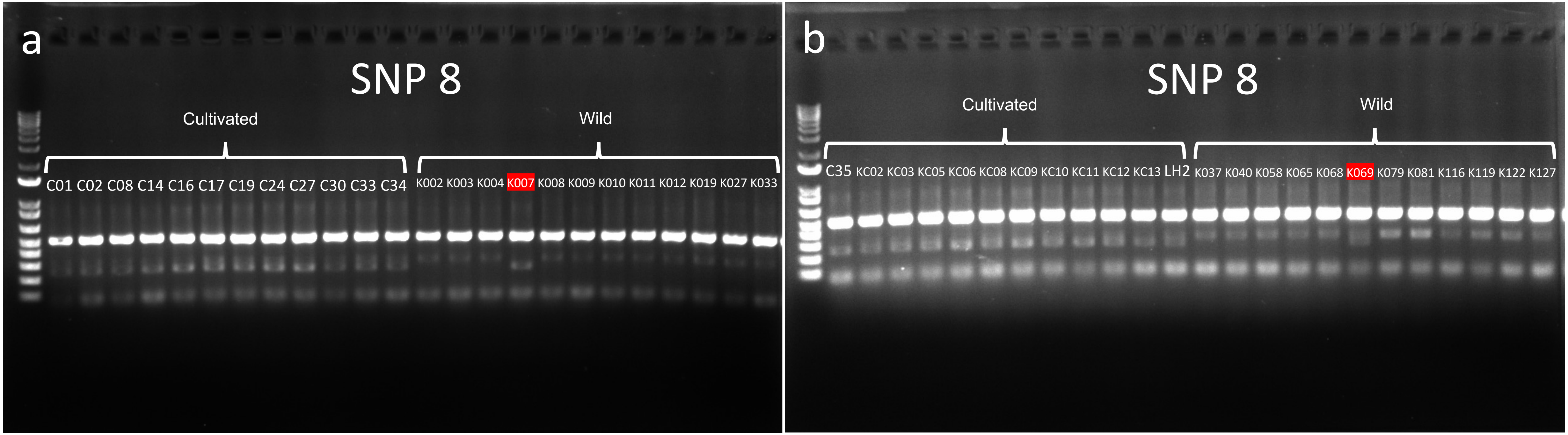

Figure S7 Agarose gel images of the results of SNP 8 testing on 48 soybean germplasms by PCR. a Twenty-four wild and cultivated germplasms. **b** Twenty-four wild and cultivated germplasms. Accessions with different genotypes in the population were highlighted in red.

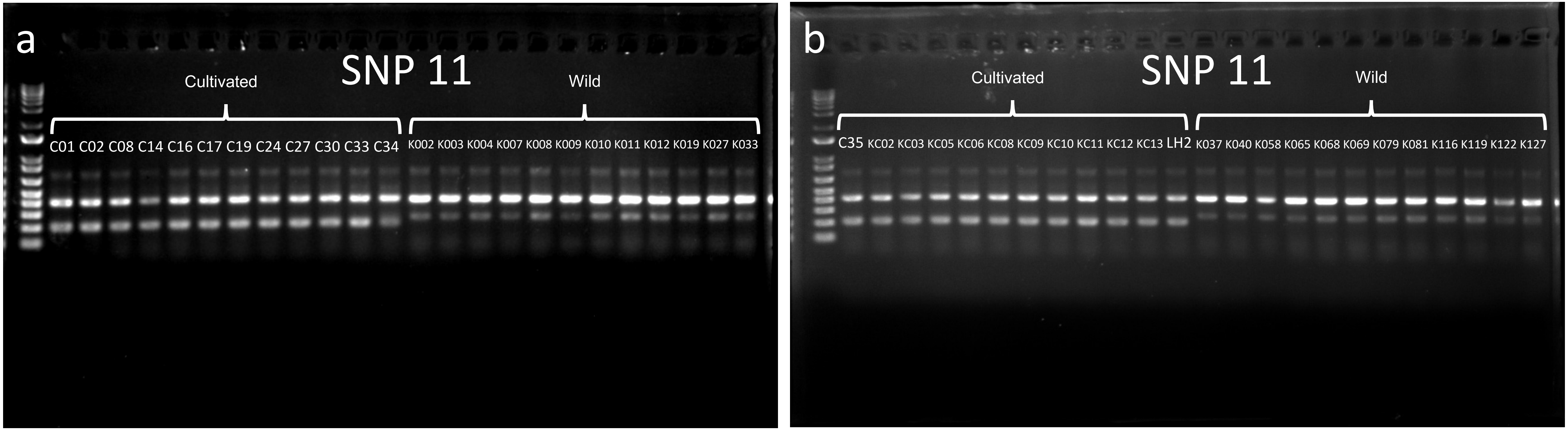

Figure S8 Agarose gel images of the results of SNP 11 testing on 48 soybean germplasms by PCR. a Twenty-four wild and cultivated germplasms. **b** Twenty-four wild and cultivated germplasms.

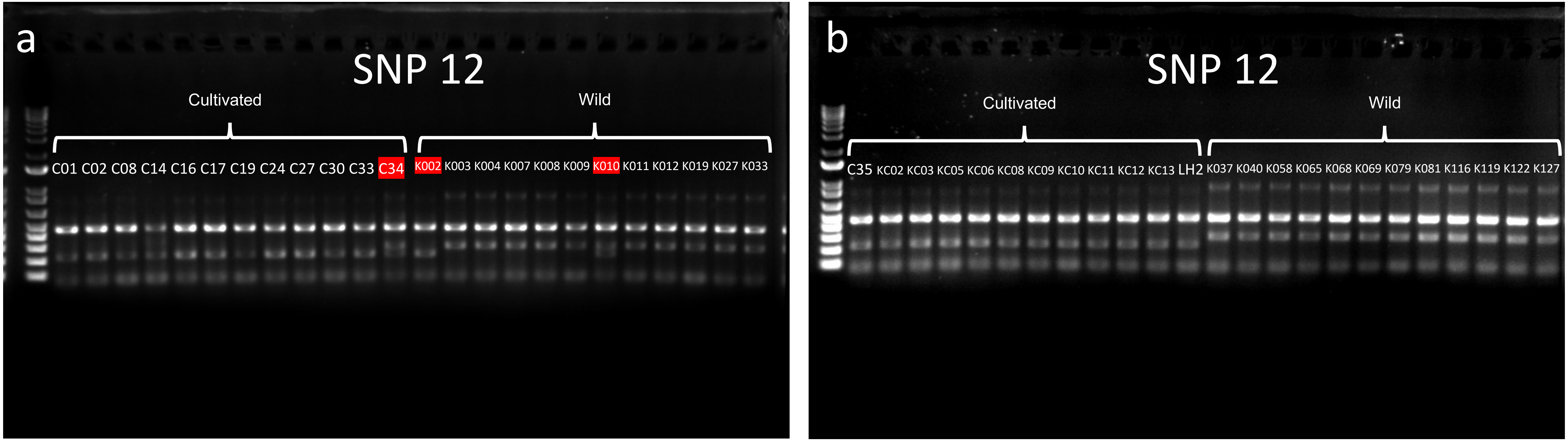

Figure S9 Agarose gel images of the results of SNP 12 testing on 48 soybean germplasms. a Twenty-four wild and cultivated germplasms. **b** Twenty-four wild and cultivated germplasms. Accessions with different genotypes in the population were highlighted in red.

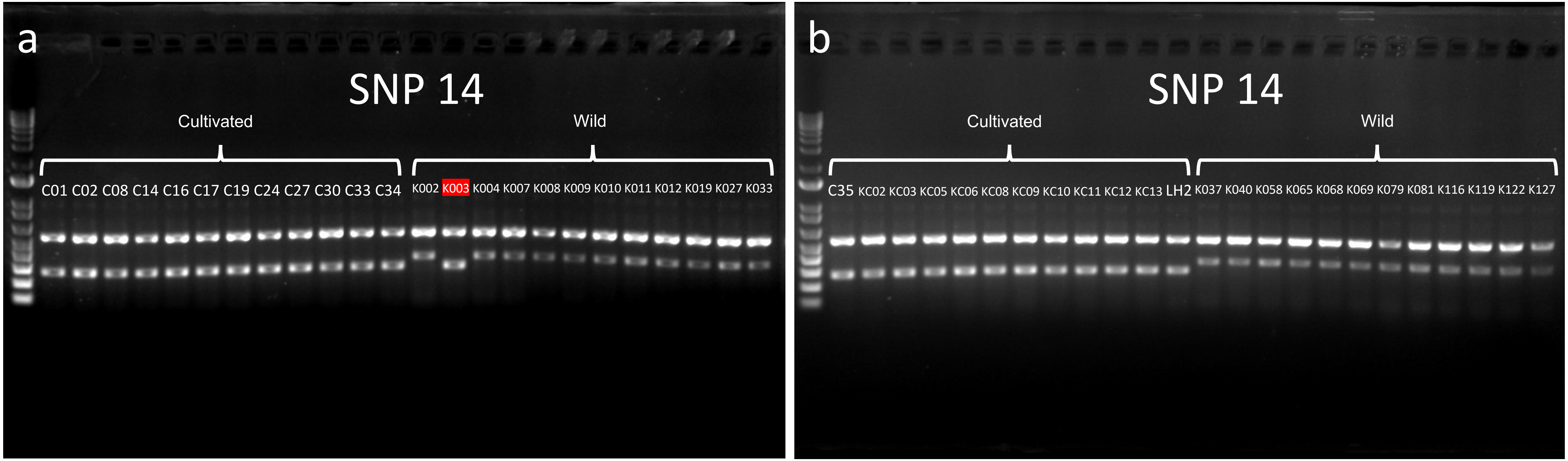

Figure S10 Agarose gel images of the results of SNP 14 testing on 48 soybean germplasms. a Twenty-four wild and cultivated germplasms. **b** Twenty-four wild and cultivated germplasms. Accessions with different genotypes in the population were highlighted in red.

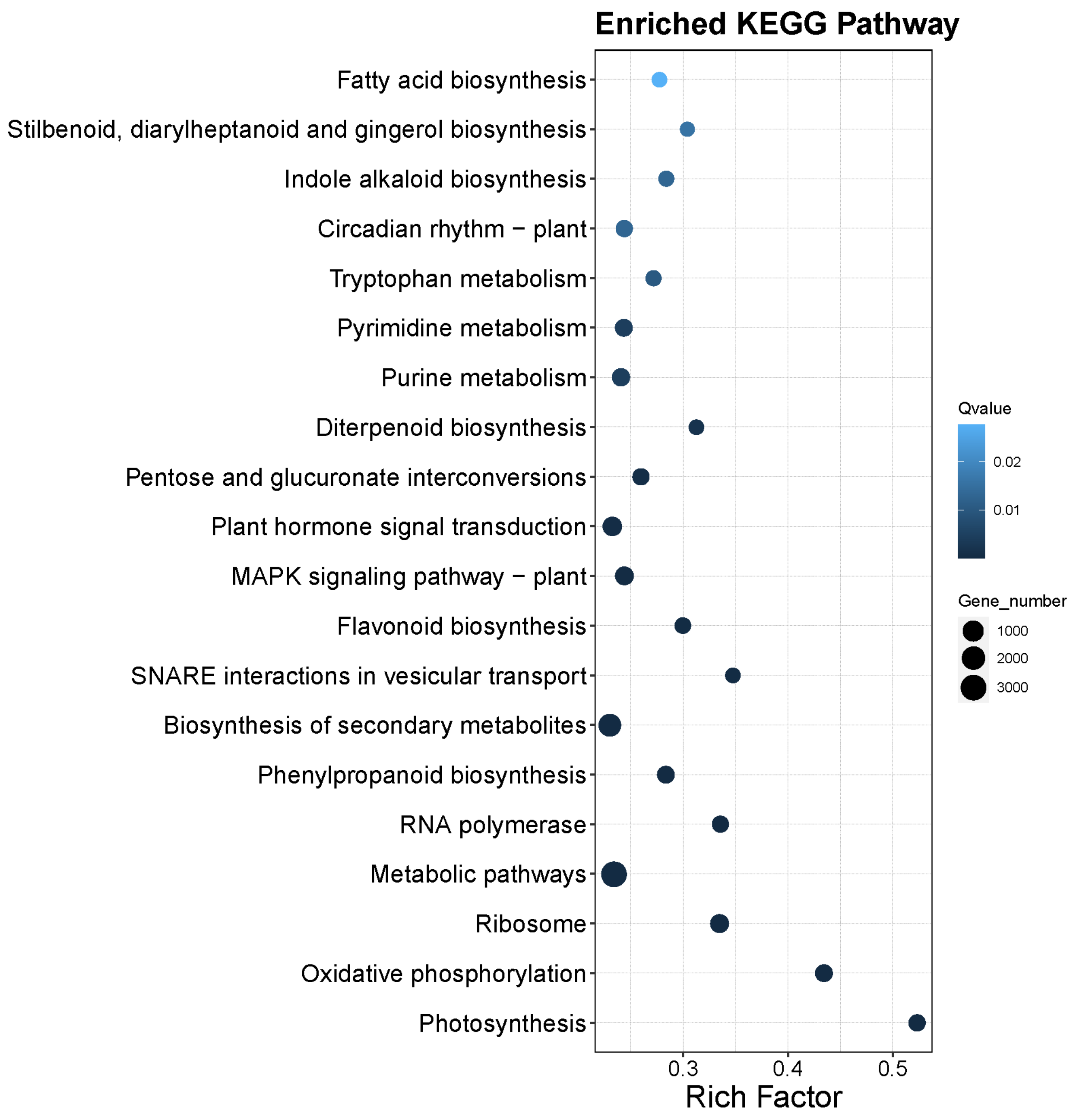

Figure S11 Enriched KEGG pathways of non-LEM genes.

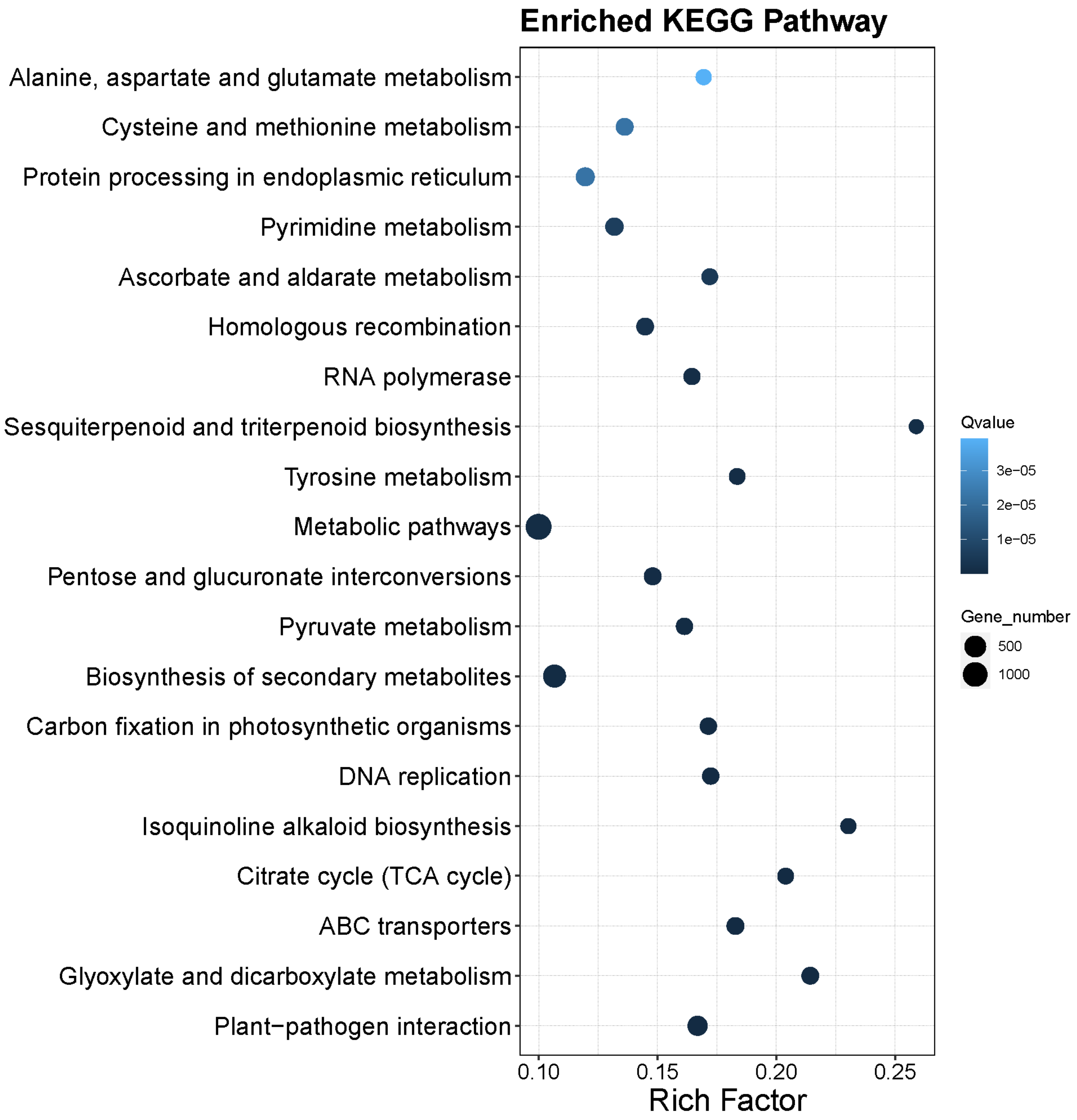

Figure S12 Enriched KEGG pathways of LEM genes with MAF ≥ 0.01.
